# Optimization of Demineralization and Pyrolysis Performance of Eucalyptus Hydrothermal Pretreatment

**DOI:** 10.3390/polym14071333

**Published:** 2022-03-25

**Authors:** Jiatian Zhu, Yuqi Bao, Luxiong Lv, Fanyan Zeng, Dasong Du, Chen Liang, Jiayan Ge, Shuangfei Wang, Shuangquan Yao

**Affiliations:** Guangxi Key Laboratory of Clean Pulp & Papermaking and Pollution Control, School of Light Industrial and Food Engineering, Guangxi University, Nanning 530004, China; 2016301053@st.gxu.edu.cn (J.Z.); lb04100126@163.com (Y.B.); luxionglv@163.com (L.L.); 2116301005@st.gxu.edu.cn (F.Z.); ggbond19114788464@163.com (D.D.); liangchen@gxu.edu.cn (C.L.); hebo1104@outlook.com (J.G.); liubaojie@st.gxu.edu.cn (S.W.)

**Keywords:** eucalyptus, demineralization, hydrothermal pretreatment, thermostability, pyrolysis products

## Abstract

The preparation of bio-oil through biomass pyrolysis is promoted by different demineralization processes to remove alkali and alkaline earth metal elements (AAEMs). In this study, the hydrothermal pretreatment demineralization was optimized by the response surface method. The pretreatment temperature, time and pH were the response elements, and the total dissolution rates of potassium, calcium and magnesium were the response values. The interactions of response factors for AAEMs removal were analyzed. The interaction between temperature and time was significant. The optimal AAEMs removal process was obtained with a reaction temperature of 172.98 °C, time of 59.77 min, and pH of 3.01. The optimal dissolution rate of AAEMs was 47.59%. The thermal stability of eucalyptus with and without pretreatment was analyzed by TGA. The hydrothermal pretreatment samples exhibit higher thermostability. The composition and distribution of pyrolysis products of different samples were analyzed by Py-GC/MS. The results showed that the content of sugars and high-quality bio-oil (C6, C7, C8 and C9) were 60.74% and 80.99%, respectively, by hydrothermal pretreatment. These results show that the removal of AAEMs through hydrothermal pretreatment not only improves the yield of bio-oil, but also improves the quality of bio-oil and promotes an upgrade in the quality of bio-oil.

## 1. Introduction

The global demand for fossil-fuel-derived energy has increased significantly with rapid economic and global population growth [1,2]. Therefore, the search for alternative energy sources presents a global challenge [3,4]. Biomass can be converted into liquid fuels or chemicals, thus effectively relieving the energy crisis and environmental pressure [5,6,7]. The pyrolysis of biomass to produce bio-oil represents one of the main methods to utilize biomass resources [8]. However, there are many technical difficulties in the fast pyrolysis process, which limits its popularization and application. Typical limitations include poor volatility, poor thermal stability, high viscosity, and low calorific value of bio-oil [9,10]. Bio-oil quality is mainly affected by the physicochemical structure of the biomass and the pyrolysis conditions [11]. In particular, alkali and alkaline earth metals (AAEMs) comprise important components of biomass, and they have a significant effect on the pyrolysis reaction [12,13]. The formation of carbonyl compounds, water, and acids are improved by the presence of AAEMs, which act as catalysts. However, this leads to a reduction in bio-oil production [14].

AAEMs in biomass can be removed by demineralization [15,16]. There are two main demineralization methods: water washing and acid leaching [17]. Although water washing can effectively remove water-soluble AAEMs, the removal of acid-soluble AAEMs is inefficient. Chen et al. [14] reported that the removal rates of water-soluble AAEMs, such as potassium in cotton stalks reached more than 80% by water washing. However, the removal rates of acid-soluble AAEMs, such as calcium and magnesium, measured 29% and 48%, respectively. On the other hand, acid leaching is very effective in removing alkaline earth metals. Ma et al. [18] studied the effect of acid leaching on the rapid pyrolysis of rice husk to produce bio-oil. The results showed that acid leaching could effectively enhance the removal of alkaline earth metals. Meanwhile, acid leaching can effectively improve the yield of bio-oil produced by rapid pyrolysis. However, some functional groups in biomass can also be damaged. Dong et al. [19] found that the hydrogen bonds in the chemical structure of the biomass were broken. In addition, the hemicellulose in the biomass is removed in large quantities. In addition, it has been reported that the structure of lignin and cellulose is also damaged, and the crystallinity is reduced. In contrast, hydrothermal pretreatment can effectively remove AAEMs while giving the biomass higher cellulose crystallinity and thermal stability [20]. Chang et al. [21] studied the effect of hydrothermal pretreatment on the rapid pyrolysis of biomass to produce bio-oil. The results showed that temperature had a significant effect on the removal of AAEMs. However, the dissolution of cellulose, hemicellulose and lignin increased with the increase of temperature. In our previous study, it was discovered that the main influencing factors of hydrothermal pretreatment were temperature, pH and time [22]. However, there are few reports on the interaction between different factors during hydrothermal pretreatment. In addition, previous studies have found that when the AAEMs removal rate of eucalyptus is equal between hydrothermal pretreatment and hydrochloric acid leaching, the yield and composition distribution of bio-oil produced by rapid pyrolysis of the biomass treated by the two pretreatment methods are different. Moreover, the forms of AAEMs in biomass may affect the yield and composition distribution of bio-oil [23]. Therefore, it is of great significance to study the dissolution rule of AAEMs in different forms during pretreatment to control the yield and component distribution of bio-oil.

In this study, the demineralization of the eucalyptus wood by hydrothermal pretreatment was optimized using the response surface method. A quadratic polynomial mathematical model of the AAEMs dissolution rate was established using temperature, pH and time as response factors and the AAMEs dissolution rate as response values. The changes in AAEMs (potassium, calcium and magnesium) content were analyzed by inductively coupled plasma atomic emission spectrometry (ICP-OES). The thermal stability of samples was analyzed by a thermogravimetric analyzer (TGA) and pyrolysis–gas chromatography combined instrument (Py-GC/MS). The chemical composition of the eucalyptus before and after pretreatment was determined by sulfuric acid hydrolysis according to the NREL method. The contents of AAEMs in different forms were analyzed by chemical fractionation analysis (CFA). The removal ability of different AAEMs by hydrothermal pretreatment and acid pickling was compared. This work aims to evaluate the demineralization of the two pretreatments on eucalyptus. The composition and distribution of pyrolysis products of eucalyptus via two pretreatments was analyzed.

## 2. Materials and Methods

### 2.1. Materials

Eucalyptus chips (20 mm × 5 mm) were provided by a local company (Guangxi, China). The chemical composition of the eucalyptus were determined by the sulfuric acid hydrolysis according to the NREL method [22]. The contents of cellulose, hemicellulose and lignin and ash from eucalyptus were 49.55%, 12.93%, 34.53% and 0.35%, respectively. Potassium, calcium, and magnesium standard solutions were purchased from Agilent Technologies, Inc. (Santa Clara, CA, USA). These analytical chemicals were purchased from Aladdin (Shanghai, China).

### 2.2. Demineralization

Hydrothermal pretreatment demineralization was carried out in a rotary digester with six stainless steel cylindrical reactors (Green Wood, Brooklyn, NY, USA). The solid–liquid ratio was 1:6. Hydrothermal pretreatment was performed at different temperatures, times, and pH values. The hydrolysate was collected and centrifuged at 10,000 rpm for 10 min after reaction. The supernatant was filtered using a 0.45 um filtration membrane to obtain the filtrate liquid. The hydrolytic solution after membrane separation was cryopreserved [24]. The demineralization of acid leaching was analyzed. The method was described in the previous study [22].

### 2.3. Sample Preparation by Microwave-Assisted Digestion 

A 1 mL volume of hydrolysate was pipetted into the polytetrafluoroethylene microwave digestion tube, and 5 mL of concentrated nitric acid and 2 mL of hydrogen peroxide were added. After the mixture was completely mixed for 15 min, the samples were digested in a microwave digestion system for 3 min at 220 °C, and cooled for 2 h. The samples were diluted for analysis [25].

### 2.4. ICP-OES Measurements

The standard solutions of potassium, calcium and magnesium were each accurately absorbed into and gradually diluted with 5% dilute nitric acid solution to configure a series of standard solutions at concentrations: 0 mg·L^−1^, 0.5 mg·L^−1^, 5 mg·L^−1^, 10 mg·L^−1^, and 20 mg·L^−1^. The standard curves for different metal ions were plotted by ICP-OES (Optima 5300 DV, Agilent Technologies Inc., Santa Clara, CA, USA) [26]. The concentration of AAEMs in the sample was detected.

### 2.5. Process Optimization 

Based on previous studies [22], the dissolution rate of AAEMs was most affected by the reaction temperature, time and pH. Therefore, the above three reaction conditions and the total dissolution rate of the three AAEMs (potassium, calcium, and magnesium) were taken as the response factors and response values, respectively. Three-factor, three-level response surface optimization experiments were designed (Table 1). The interaction between different factors was studied.

### 2.6. Component Analysis

The main components of the samples with and without pretreatment were analyzed. First, 40–60 mesh wood powder was obtained by screening. Then, 20 g of the powder was reacted with benzyl alcohol for 8 h, and later subjected to a two-step acidolysis process. The specific methods and processes used were demonstrated by Ge and co-authors [22]. The relative contents of cellulose, hemicellulose and lignin were analyzed [27].

### 2.7. Chemical Fractionation

**Figure 1:**
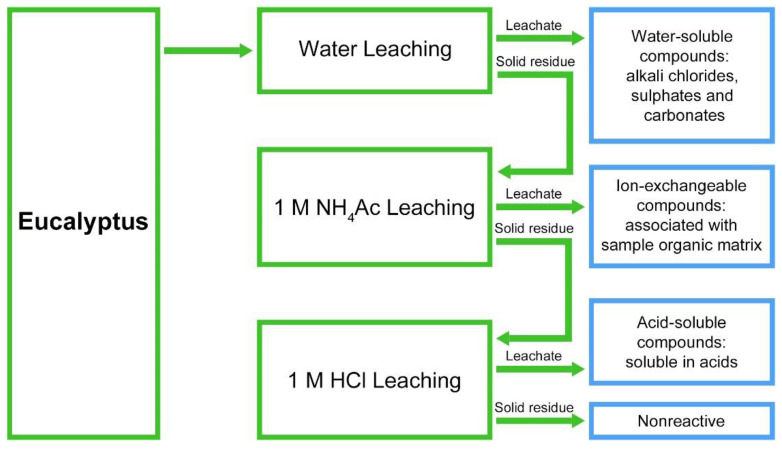
Chemical fractionation general procedure.

The chemical fractionation was carried out according to Pettersson and co-authors [28]. It is a step-by-step leaching method resulting in selective extraction of inorganic elements, based on the solubility of their association forms in the samples (Figure 1). The experimental procedure consists of three successive extractions. First, the water-soluble compounds such as alkaline salts are removed using pure water. Then, addition of ammonium acetate dissolves ion exchangeable elements, such as sodium, calcium and magnesium. The third extraction step with hydrochloric acid removes acid soluble compounds. The solid residue fraction consists of silicates, oxides, sulphides and other minerals. After each step the solid sample was washed two times by deionized water. The washing water was added to the leachate prior to analysis.

### 2.8. Rapid Pyrolysis of Eucalyptus

Eucalyptus samples with and without pretreatment were crushed by a grinder. The sawdust with a particle size range of 30–80 mesh was selected for the rapid pyrolysis reaction. The pyrolysis reaction was carried out by a small, fixed bed pyrolysis device built in the laboratory. High purity nitrogen (500 mL·min^−1^) was continuously injected to provide an inert environment for pyrolysis, and 10 g samples were selected for each pyrolysis experiment. The pyrolysis temperature was 500 °C, and the pyrolysis time was 10 min. The pyrolysis gas product (non-condensable gas) was collected with a collector bag. The liquid product (bio-oil) was collected by a condenser directly connected to the pyrolysis reactor. The solid product (biochar) remained in the reactor [29].

### 2.9. Pyrolysis Performance Characterization

The thermo-gravimetric analysis (TG) and differential thermal gravity (DTG) of the samples were analyzed via thermal-gravimetric analyzer (STA 449 F5 Jupiter, Netzsch, Germany). A 10.0 mg mass of the sample was placed in an alumina crucible at nitrogen atmosphere. The temperature was increased from 30 °C to 800 °C at 10 °C·min^−1^ [30].

The yield of bio-oil and biochar was calculated by the differential method. The gas velocity was calculated from the gas pressure drop value inside the fixed bed. The difference between the gas velocity and carrier gas velocity in the fixed bed provided the velocity of pyrolysis gas. The amount and yield of non-condensable gas was calculated [31]. The water content of bio-oil was measured using Karl Fischer hydrometer (KF DL31, Mettler-Toledo, Zurich, Switzerland). The viscosity of bio-oil was measured by ChemTron Viscolead rotational viscometer (ChemTron, Celle, Germany) [32]. The chemical composition and distribution of bio-oil was determined by Py-GC/MS. Pyrolysis was performed at 550 °C. Analytical Py-GC/MS experiments were performed using a pyrolysis furnace (a VF-1701 MS column) connected to the Agilent 7890 A gas chromatograph. The basic method and process were described by Gu and co-authors [33].

## 3. Results and Discussion

### 3.1. Response Surface Design and Results

Box–Behnken was used for evaluating the effect of concentration of reaction temperature (x_1_), time (x_2_), pH (x_3_) on the total dissolution rate of AAEMs. The experimental design and results are shown in Table 2.

The experimental data were analyzed by regression. The responses and independent variables were correlated by the resulting second-order polynomial Equation (1).

Y(%) = 41.73 + 2.10x_1_ − 0.29x_2_ − 5.63x_3_ + 0.61x_1_x_2_ − 0.39x_1_x_3_ − 0.41x_2_x_3_ − 3.29x_1_^2^ − 2.73x_2_^2^ − 0.16x_3_^2^ (1)

### 3.2. Interaction between Reaction Factors

**Figure 2:**
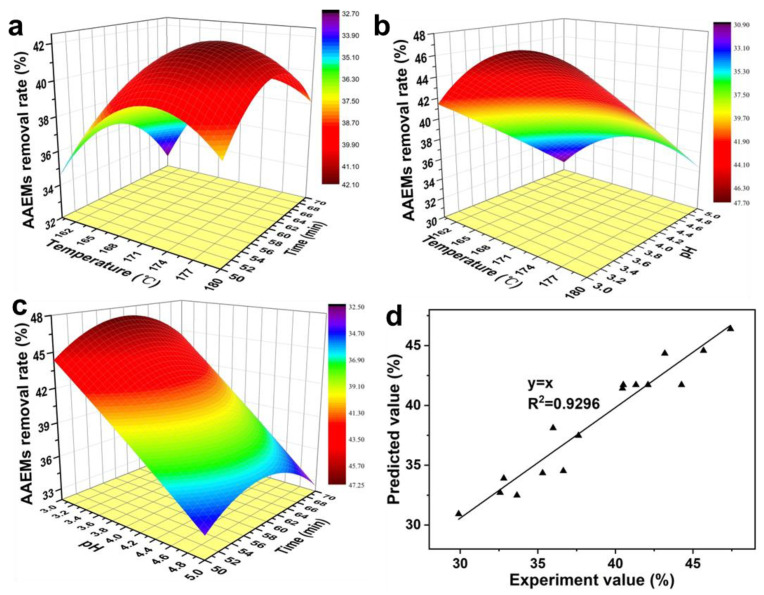
Interaction of reaction temperature, time and pH on the total removal rate of AAEMs. ((**a**), interaction between temperature and time on the total removal rate of AAEMs. (**b**), interaction between temperature and pH on the total removal rate of AAEMs. (**c**), interaction between pH and time on the total removal rate of AAEMs. (**d**), fitting relationship between the predicted value and the experiment value).

A change in the color of the 3D response surface graph from blue to red indicates a change in the extraction quality from less to more. The faster the change, the greater the slope, and the more significant the impression of the test result. The optimum process parameters and the interaction between the parameters were studied. The results are shown in Figure 2.

Figure 2a shows the interaction between reaction temperature and holding time on the total removal rate of AAEMs at a fixed pH 4.0. The total removal rate of AAEMs varies with time with a similar change rule at different temperatures, increasing with time from 50 min to 60 min. However, it decreases with time from 60 min to 70 min. This is due to the fact that the dissolution of AAEMs was promoted with increased cell wall damage as the reaction progressed [34]. At the same time, carbohydrate degradation increased with reaction time [35,36]. The formation of organic acids was promoted. The complexation reaction between AAEMs and organic acids was intensified with the increase of organic acid concentration [37]. The dissolution of AAEMs was inhibited by intracellular accumulation of complexes. The results also show that the variation range of the total removal rate of AAEMs decreases within the same temperature range with the increase of temperature. The total AAEMs removal rate at 160 °C for 50 min and 70 min measured 36.65% and 32.57%, respectively. It was 37.62% and 35.99% at 180 °C for 50 min and 70 min. The degree of cell wall damage increases with increasing temperature. The removal rate of AAEMs increased at the same time. The formation of organic acids was facilitated by the increased acidity of hydrolysates at high temperatures. However, the residual AAEMs in the cell were reduced due to increased damage to the cell wall. The complexation reaction between AAEMs and organic acids was reduced. Compared with low temperature, the total removal rate of AAEMs increased. The results showed that temperature and time exhibited a significant interaction effect on the removal of AAEMs.

The interactive influence of reaction temperature and pH value on the total removal rate of AAEMs at fixed reaction time (60 min) is shown in Figure 2b. The effect of pH on AAEMs removal was similar at different temperatures. The total removal rate of AAEMs increased with an increase of pH at low pH values (3.0–4.0). However, the total removal rate of AAEMs decreased with pH values between 4 and 5. This is due to the extent of cell wall damage being exacerbated by strong acidity [38]. Specifically, the AAEMs removal rates of pH 3 and 5 were 40.46% and 29.90%, respectively, at 160 °C. However, the removal effect of AAEMs was influenced by the complexation of AAEMs and organic acids inside the cell. Previous studies have shown that the optimal extraction of hemicellulose was obtained during hydrothermal pretreatment at pH 4 [39]. This means that the maximum cellulose extraction yield was obtained while the degradation was inhibited. This results in a decrease in organic acid content. The degree of complexation reaction between AAEMs and organic acid was reduced. Under the interaction of cell wall rupture and organic acid complexation reaction, AAEMs removal rate was higher at pH 4. Figure 2b also shows that the total removal rate of AAEMs increases with an increase of temperature at the same pH. The AAEMs removal rates of pH 3 and 5 were 47.42% and 35.31%, respectively, at 180 °C. This is because the acidity of the hydrolysate and the steam pressure increase with increasing temperature [40]. The damage to the cell wall was exacerbated and the removal of AAEMs was facilitated. The results showed that pH was dominant in the interaction between pH and temperature on AAEMs removal.

Figure 2c shows the interaction of pH and time with AAEMs removal at a fixed temperature (170 °C). The effect of time on AAEMs removal was similar to the effect of time on AAEMs removal in Figure 2a. In addition, the removal rate of AAEMs decreases with an increase of pH at the same time. The reasons for this have been explained above. Different from Figure 2b, AAEMs removal rate was not abnormal at pH 4. This is because the consumption of AAEMs in the complexation reaction was much lower than the total amount of AAEMs released by the cells at 170 °C. At this point, the removal of AAEMs was mainly affected by the amount of AAEMs dissolution after cell wall rupture. The above results indicate that pH dominates the interaction between time and pH on AAEMs removal.

The optimal process of AAEMs removal in hydrothermal pretreatment was obtained by response surface design, namely at a temperature of 172.98 °C, time of 59.77 min, and pH of 3.01. The optimal AAEMs total removal rate was 47.59%. In addition, the accuracy of the response surface model was analyzed. The results are shown in Figure 2b. There is a good linear correlation between the experimental value and the predicted value. The correlation coefficient R^2^ of the linear regression equation between them was 0.9296. This means that the model exhibits a high degree of accuracy. The prediction data of the model are real and effective.

### 3.3. Thermal Stability Analysis

**Figure 3:**
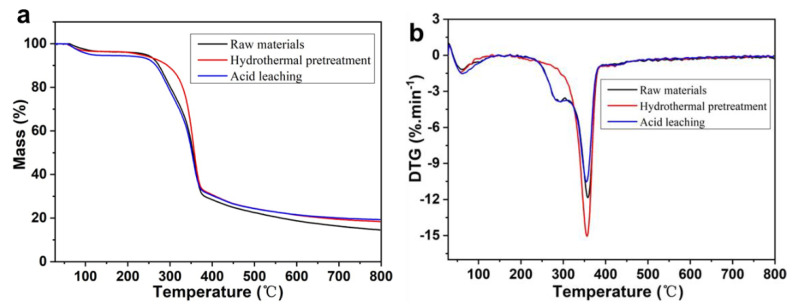
TG (**a**) and DTG (**b**) of eucalyptus with or without acid leaching and hydrothermal pretreatment.

The thermal stability of woody biomass improved with the removal of AAEMs. The rapid pyrolysis of biomass was promoted. High quality and high yield of bio-oil was obtained [41]. Thermo-gravimetric analysis (TGA) of eucalyptus during hydrothermal pretreatment and acid leaching was studied and compared at the same removal rate of AAEMs. The results are shown in Figure 3.

There are several significant changes in Figure 3a. The first is that the initial decomposition temperature of the sample is different. The initial decomposition temperatures of raw materials and acid leaching samples were 254 °C and 255 °C, respectively. Remarkably, the initial decomposition temperature of the hydrothermal pretreatment sample significantly increased to 296 °C. More importantly, the maximum weight loss of the samples differed although their final decomposition temperature was similar at 377 °C. The maximum weight loss of raw material, acid leaching and hydrothermal pretreatment samples was 69.49%, 67.47% and 66.90%, respectively. As is well known, the effect of acid leaching on the physicochemical structure of woody biomass was significant. The dissolution and degradation of cellulose, hemicellulose and lignin was improved at the same removal effect of AAEMs. This resulted in a reduction of the number of decomposable components. The maximum weight loss of acid leaching sample was reduced. However, AAEMs were effectively removed while hemicellulose was selectively removed during hydrothermal pretreatment. The initial decomposition temperature was increased due to the decrease of hemicellulose content after pretreatment. Correspondingly, the relative contents of cellulose and lignin in the pretreated sample was increased. Thus, it also bears a higher residual mass than the raw material. The results show that the hydrothermal pretreated sample has higher initial decomposition temperature. However, this is insufficient to indicate an improvement in thermal stability, and is also related to the maximum rate of weight loss.

The maximum weight loss rates of different samples are shown in Figure 3b. The maximum weight loss rate of raw materials was 11.85 %·min^−1^. It decreased to 10.53 %·min^−1^ with acid leaching. This indicates that the removal of AAEMs is accompanied by the loss of more effective pyrolysis components (cellulose). This is inconducive to the thermal and chemical utilization of woody biomass. Contrary to acid leaching, hydrothermal pretreated samples exhibit a higher maximum weight loss rate (15.06 %·min^−1^). This means that the sample has higher thermal stability. In addition, the DTG curves of different samples provide another important piece of information. The “shoulder peak” exists in the DTG curve of raw materials and acid leaching samples. It is a significant marker of the presence of hemicellulose in samples. However, there was no such peak in the sample after hydrothermal pretreatment. This also verifies our previous inference that hydrothermal pretreatment can efficiently remove AAEMs while selectively removing hemicellulose. The results showed that the hydrothermal pretreated sample exhibits higher thermal stability than the acid leaching sample at the same removal effect of AAEMs.

### 3.4. Pyrolysis Performance Analysis

It is well known that the pyrolysis properties and products of cellulose, hemicellulose and lignin differ. Therefore, the effect of different pretreatments on the fast pyrolysis performance of eucalyptus was studied. The results are shown in Table 3.

Table 3 shows that the yield of bio-oil was increased by acid leaching. The yield of biochar and non-condensable gas was decreased. This is consistent with previous research [42,43]. Significantly, a higher yield of bio-oil was obtained by hydrothermal pretreatment demineralization, while the generation of biochar and non-condensable gas was inhibited. A large amount of hemicellulose was selectively removed during hydrothermal pretreatment [36,39]. The pyrolysis reaction was facilitated by the higher cellulose and lignin content in the sample. In addition, the biomass pyrolysis reaction was affected by the synergies between the three components (cellulose, hemicellulose and lignin) [44]. The synergistic effect of hemicellulose and cellulose was not evident. The synergetic effect of cellulose and lignin pyrolysis was conspicuous [45]. The formation of laevoglucose was inhibited during cellulose pyrolysis due to the presence of lignin. Therefore, the formation of low molecular weight products was promoted, and the yield of biochar was reduced. The formation of secondary carbon products during lignin pyrolysis was inhibited, and the formation of lignin pyrolysis products, such as o-methoxyphenol and 4-methyl guaiacol, was promoted due to the presence of cellulose. Meanwhile, the gas products (CO, H_2_, CH_4_ and C_2_H_4_) were clearly inhibited by the synergistic effect between cellulose and lignin. Based on the above research conclusions, the synergistic effect of cellulose and lignin in the samples after hydrothermal pretreatment further promoted the yield of bio-oil and decreased the yield of biochar and non-condensable gas.

The effects of acid leaching and hydrothermal pretreatment on moisture in bio-oil are shown in Table 3. The moisture in bio-oil with acid leaching decreases to 5.94%. However, the moisture reduction effect with hydrothermal pretreatment was more significant, measured at 7.54%. In fact, the pyrolysis water mainly originates from the dehydration reaction of the structural units in cellulose and hemicellulose [46]. For example, ketones and ethers were produced by the dehydration reactions between adjacent cellulose chains. In addition, the pyrolysis water was generated from the dehydration of lignin molecules. The content of pyrolysis water formed by dehydration reaction of hemicellulose was greatly reduced, which was due to the selective removal of hemicellulose by hydrothermal pretreatment.

Table 3 shows that a higher concentration of bio-oil was obtained by acid leaching and hydrothermal pretreatment. The viscosity of bio-oil with acid leaching was higher under the same removal capacity of AAEMs. This was due to an increase in its bio-oil “superheavy components” (solids that do not decompose by heat). As shown in Figure 3, the thermal stability of solid residues in the sample with acid leaching was higher than that of the corresponding components in the sample with untreated or hydrothermal pretreatment.

### 3.5. Chemical Composition and Distribution of Bio-Oil

**Figure 4:**
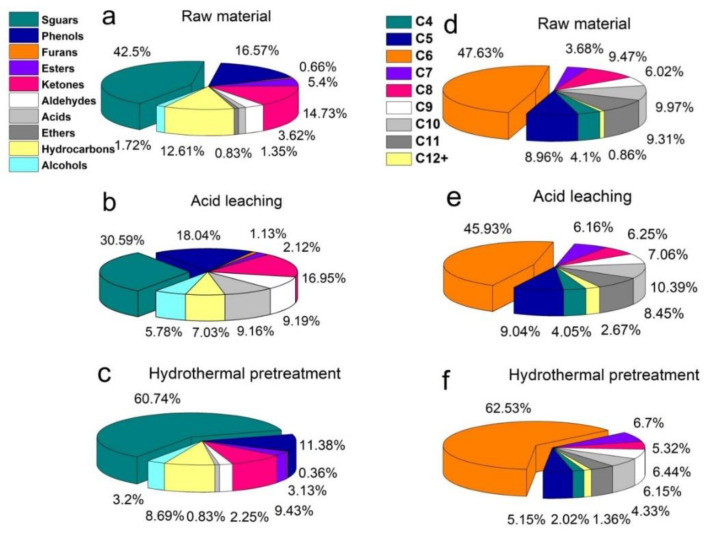
Composition and distribution of pyrolytic bio-oil from different eucalyptus samples (**a**–**c**), components of pyrolytic bio-oil from raw material, acid leaching eucalyptus and hydrothermal pretreatment eucalyptus. (**d**–**f**), distribution of pyrolytic bio-oil from raw material, acid leaching eucalyptus and hydrothermal pretreatment eucalyptus.

The chemical composition and distribution of bio-oils are altered by demineralization [47]. Figure 4a shows that the bio-oils from raw material samples were mainly composed of sugars, phenols, ketones and hydrocarbons. The contents were 42.50%, 16.57%, 14.73% and 12.61%, respectively. The sugars of bio-oil decreased to 30.59% after acid leaching (Figure 4b). This was attributed to changes in the composition of eucalyptus during acid leaching for AAEMs removal. Table 4 shows the contents of cellulose, hemicellulose and lignin in eucalyptus after acid leaching decreased to 37.55%, 9.68% and 16.54%, respectively. In fact, high yield of laevoglucose is obtained in the pyrolysis of cellulose [48]. The pyrolysis products of hemicellulose mainly include hydrocarbons, acids and ketones [49]. Phenols are obtained from lignin pyrolysis [50]. Cellulose, hemicellulose and lignin were significantly degraded by acid leaching. Therefore, the content of main components of bio-oil was reduced. In addition, hydrothermal pretreatment has little effect on the composition of eucalyptus. Hemicellulose was selectively removed (Table 4). Therefore, the sugars increased to 60.74% after hydrothermal pretreatment (Figure 4c). The bio-oils from raw material samples are mainly divided into high-quality bio-oils (C6, C7, C8 and C9), light bio-oils (C4 and C5) and heavy bio-oils (C10, C11 and C12+), as shown in Figure 4d. The contents were 66.80%, 13.06% and 20.14%, respectively. The content of high-quality bio-oil decreased to 65.40% after acid leaching (Figure 4e). Its content increased to 80.99% in the hydrothermal pretreatment sample (Figure 4f). This was due to the protection of cellulose. The content of acids and ketones in hydrothermal pretreatment sample was reduced to 0.83% and 9.43%, respectively. This reduces the light bio-oil content of the hydrothermal pretreatment sample to 7.17%. Therefore, the quality of bio-oil was improved by hydrothermal pretreatment.

**Figure 5:**
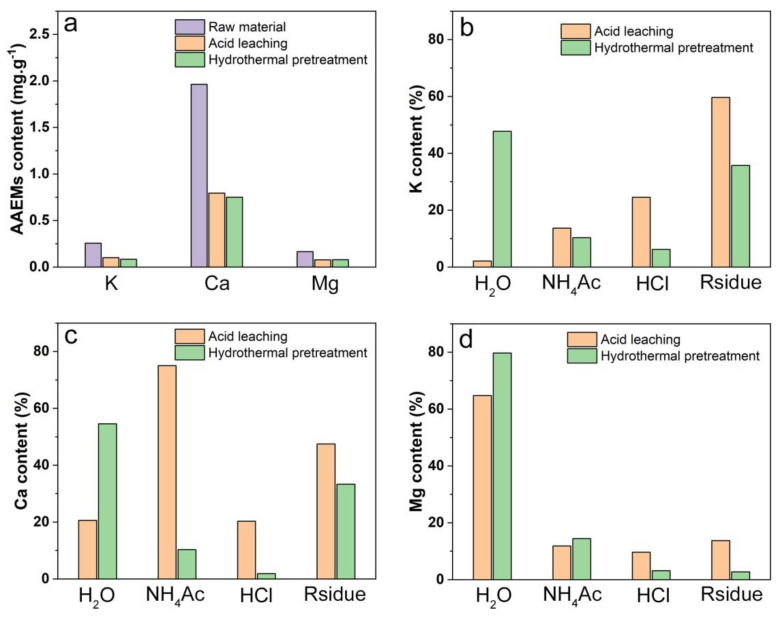
Forms of main metal elements in eucalyptus before and after hydrothermal pretreatment and acid leaching ((**a**), contents of K, Ca and Mg in different eucalyptus samples. (**b**), contents of K in different forms in eucalyptus after acid pickling and hydrothermal pretreatment. (**c**), contents of Ca in different forms in eucalyptus after acid pickling and hydrothermal pretreatment. (**d**), contents of Mg in different forms in eucalyptus after acid pickling and hydrothermal pretreatment).

Table 4 shows that the lignin content of hydrothermal pretreatment sample was higher than that of acid leaching sample. However, the concentration of phenols in the hydrothermal pretreatment sample were low, measured at 11.38% (Figure 4c). This means that there are other factors affecting the composition and distribution of bio-oil. The content of AAEMs in different forms was changed by demineralization [51]. In fact, hydrothermal pretreatment and acid leaching exert different effects on the dissolution of AAEMs in different forms. Therefore, different forms of AAEMs in eucalyptus after hydrothermal pretreatment and acid leaching were analyzed. Figure 5a shows that the content of potassium, calcium, and magnesium were the same in eucalyptus after different treatments. This verifies that acid leaching and hydrothermal pretreatment exhibit the same AAEMs removal rate. However, more water-soluble AAEMs were obtained in hydrothermal pretreatment sample (Figure 5b–d). The deoxidation of lignin during pyrolysis was promoted to utilize water-soluble AAEMs [52]. Therefore, the yield of phenols in hydrothermal pretreatment samples were reduced. In addition, Figure 5c shows that acid-soluble Ca^2+^ content was higher in acid leaching sample. Calcium carboxylate was formed as a result of acid-soluble Ca^2+^ binding to esters during pyrolysis. The yield of ester products was reduced [53]. In addition, the yield of ketones was increased by further decomposition of calcium carboxylate into linear ketones [54]. Decreased lipid content and increased ketones in hydrothermal pretreatment samples were explained. This indicates that biomass was effectively protected during hydrothermal pretreatment, and more water-soluble AAEMs were retained. The pyrolysis efficiency of biomass showed improvement and higher quality bio-oils were obtained.

## 4. Conclusions

The interaction of temperature, time, and pH value on AAEMs removal during hydrothermal pretreatment was studied by the response surface method. The optimal AAEMs removal process and the best removal rate were obtained. Compared with acid leaching, the hydrothermal pretreated samples exhibited higher thermal stability. The content of sugars and the yield of high-quality bio-oil in the pyrolysis products were significantly increased. The results show that the hydrothermal pretreatment bears high effective demineralization and practical application value.

## Figures and Tables

**Table 1:** Response factors and design levels.

Code	Response Factor		
	Temperature (x_1_) °C	Time (x_2_) min	pH (x_3_)
−1	160	50	3
0	170	60	4
1	180	70	5

**Table 2:** Response surface experiment design and results.

Run	Factor			Response
	x_1_ (°C)	x_2_ (min)	x_3_	Total Removal Rate Y (%)
1	170	60	4	43.10
2	180	70	4	35.99
3	170	50	5	32.81
4	180	60	3	47.42
5	170	60	4	44.44
6	160	70	4	32.57
7	180	50	4	37.62
8	170	60	4	44.26
9	170	50	3	43.18
10	180	60	5	35.31
11	170	70	3	45.68
12	160	60	3	40.46
13	170	70	5	33.66
14	170	60	4	43.51
15	170	60	4	44.32
16	160	50	4	36.65
17	160	60	5	29.9

**Table 3:** Pyrolysis characteristics of eucalyptus with and without acid leaching and hydrothermal pretreatment.

Samples	Bio-Oil Yield (%)	Biochar Yield (%)	Non-Condensable Gas Yield (%)	Bio-Oil Moisture (%)	Bio-Oil Viscosity (mPa·s)
Raw material	49.56	18.24	26.43	27.08	53.17
Acid leaching	59.59	14.31	20.46	21.14	95.25
Hydrothermal	65.87	12.89	15.65	19.54	89.51

**Table 4:** Chemical composition of eucalyptus before and after hydrothermal pretreatment and acid leaching.

Samples	Cellulose (%)	Hemicellulose (%)	Lignin (%)
Raw material	49.55	14.93	32.53
Acid leaching	37.55	9.68	16.54
Hydrothermal pretreatment	47.02	6.24	33.17

## Data Availability

The data presented in this study are available in the manuscript’s figure.
